# TUBB1 promoter methylation is a promising biomarker for predicting HBeAg seroconversion in chronic hepatitis B

**DOI:** 10.1128/spectrum.01344-25

**Published:** 2025-10-09

**Authors:** Tong Zhao, Yuna Tang, Yu Sun, Jihui Li, Yuchen Fan, Chao Cui, Shuai Gao, Kai Wang

**Affiliations:** 1 Department of Hepatology, Qilu Hospital of Shandong University 91623, Jinan, China; 2 Hepatology Institute of Shandong University, Shandong University 12589, Jinan, China; 3 Qilu Hospital of Shandong University Dezhou Hospital, Dezhou, China; Institute for Biomedical Research on Retroviruses and AIDS (INBIRS), Buenos Aires, Argentina

**Keywords:** TUBB1, promoter methylation, chronic hepatitis B, HBeAg seroconversion

## Abstract

The identification of predictive indices for hepatitis B e antigen seroconversion (HBeAg SC) in patients with chronic hepatitis B (CHB) remains a challenge. We aimed to investigate whether the TUBB1 promoter methylation in peripheral blood mononuclear cells (PBMCs) can predict HBeAg SC. A total of 271 participants were recruited, comprising 145 patients with HBeAg-positive CHB, 94 with HBeAg-negative CHB, and 32 healthy controls (HCs). The patients with HBeAg-positive CHB were followed up for 72 weeks. The TUBB1 promoter methylation and the corresponding mRNA levels in PBMCs were detected using MethyLight and quantitative real-time PCR, respectively. The methylation levels of the TUBB1 promoter were remarkably elevated in patients with positive HBeAg, in comparison to those with negative HBeAg and HCs. Conversely, the relative mRNA expression levels of TUBB1 were significantly downregulated in patients with positive HBeAg, when compared to those with negative HBeAg and HCs. The TUBB1 promoter methylation levels showed a gradual decrease across the four phases of CHB. Patients with HBeAg SC had lower baseline methylation levels of the TUBB1 promoter than those without HBeAg SC. The TUBB1 promoter methylation was an independent predictor of HBeAg SC (odds ratio [OR] = 0.683, 95% CI 0.553–0.845, *P* < 0.001). The methylation of the TUBB1 promoter showed good predictive value for HBeAg SC in patients with positive HBeAg (area under the curve [AUC] = 0.805, 95% CI 0.704–0.907, *P* < 0.001). The methylation level of the TUBB1 promoter might be a potent biomarker for predicting HBeAg SC.

**IMPORTANCE:**

Previous studies emphasized hepatitis B e antigen seroconversion (HBeAg SC) as a milestone for chronic hepatitis B (CHB) remission associated with reduced disease progression risks. While the significance of HBeAg SC is widely recognized, reliable non-invasive predictors for achieving this endpoint remain limited. Additionally, in our previous studies, DNA methylation of key regulatory genes has been linked to CHB progression. However, the association between TUBB1 promoter methylation and HBeAg SC, as well as its potential as a biomarker for clinical application, has not been fully elucidated. We demonstrated that TUBB1 promoter methylation levels were significantly higher in HBeAg-positive patients and that decreased methylation levels were independently associated with subsequent HBeAg SC during a 72-week follow-up. Our findings underscore the potential clinical utility of TUBB1 promoter methylation as a non-invasive biomarker for predicting HBeAg SC. This study provides strong evidence supporting the role of TUBB1 promoter methylation in predicting HBeAg SC, offering a novel biomarker for monitoring CHB.

## INTRODUCTION

Globally, an estimated 296 million individuals are chronically infected by hepatitis B virus (HBV), and in 2022, hepatitis B was responsible for approximately 1.1 million deaths (1). Patients who are hepatitis B e antigen (HBeAg) positive with persistently elevated alanine aminotransferase (ALT) levels generally exhibit high HBV DNA concentrations, putting them at an increased risk of hepatocellular carcinoma and cirrhosis, necessitating antiviral therapy (2–7). HBeAg seroconversion (SC), defined as the clearance of HBeAg accompanied by the appearance of anti-HBe antibodies, is a key therapeutic objective. Early HBeAg SC may indicate disease remission and is associated with a favorable prognosis (8–10). Accordingly, HBeAg SC is an important goal in antiviral strategies (3–6). Therefore, a reliable and accurate non-invasive marker is urgently needed to predict early HBeAg SC, assess patient status, and guide clinical decision-making.

Methylation of cytosine phosphate-guanine (CpG) islands in deoxyribonucleic acid (DNA) is a highly prevalent epigenetic phenomenon in mammalian genomes, playing a crucial role in gene regulation. This process has been demonstrated to exert a myriad of biological effects, encompassing normal developmental processes, ribonucleic acid (RNA) metabolism, X-chromosome inactivation, genomic imprinting, and even the development of tumors (11–15). Previous studies showed that DNA methylation of key regulatory regions might be a biomarker for the progression of chronic hepatitis B (CHB), underscoring its potential role in monitoring and predicting disease advancement (16–19).

Microtubules, assembled by heterodimers of α-tubulin and β-tubulin, constitute one of the primary cytoskeletal structures in cells, playing crucial roles in maintaining cell morphology, intracellular transport, signal transduction, cell motility, and mitosis. Specifically encoded by the TUBB1 gene, β1-tubulin serves as the most prevalent isoform of β-tubulin (20–24). It was reported that TUBB1 was involved in the pathophysiology of various diseases, including thrombocytopenia, thyroid dysgenesis, neurodevelopmental defects, and so forth (25–28). TUBB1 was reported to be a potential therapeutic compound and druggable target for hepatocellular carcinoma patients (29). Besides, in the study proposed by Wang H et al., single-cell RNA sequencing revealed that TUBB1+ monocyte in peripheral blood mononuclear cells (PBMCs) might be associated with decreased antiviral activity in patients with CHB (30). However, it remains uncertain whether TUBB1 and the methylation of its promoter take part in the natural history of CHB and HBeAg SC.

In this study, we evaluated the mRNA expression levels of TUBB1 and the methylation levels of the TUBB1 promoter in PBMCs among patients with CHB and healthy controls (HCs). We observe varying levels of methylation in the TUBB1 promoter across the four phases of CHB. Our findings indicate that TUBB1 promoter methylation levels in HBeAg-positive patients independently predict HBeAg SC.

## MATERIALS AND METHODS

### Patients’ selection

A total of 271 participants, including 239 patients with CHB and 32 HCs, were recruited at Qilu Hospital of Shandong University from January 2022 to June 2023. All the patients with CHB were identified as HBsAg-positive for a minimum duration of 6 months. Exclusion criteria included: (i) coinfection with hepatitis C virus, hepatitis D virus, hepatitis E virus, or human immunodeficiency virus; (ii) combined with other liver disease (alcoholic liver disease, non-alcoholic fatty liver disease, autoimmune liver disease, drug-induced liver injury, Wilson disease); (iii) presence of hepatocellular carcinoma or other malignant disease; and (iv) pregnancy. All the subjects gave their written informed consent to participate in the study. The research was approved by the local Research and Ethics Committee at Qilu Hospital of Shandong University, in accordance with the guidelines of the 1975 Declaration of Helsinki. All experiments involving human blood samples were performed in a Biosafety Level 2 facility in accordance with institutional guidelines and regulations.

### Study design

We collected the PBMCs from all enrolled patients and HCs at baseline and extracted the DNA and mRNA to detect the TUBB1 promoter methylation and the corresponding mRNA expression. Baseline clinical and laboratory data were collected and analyzed. Patients with positive HBeAg were followed up for 72 weeks to see whether they could achieve HBeAg SC. Seroconversion was confirmed by standard assays as the simultaneous loss of HBeAg and the emergence of anti-HBe positivity.

### DNA extraction and sodium bisulfite modification

PBMCs were separated by density gradient centrifugation with Ficoll-Paque (Pharmacia Diagnostics, Uppsala, Sweden) and stored at −80°C until use. Genomic DNA was extracted from PBMCs using TRIzol Reagent (Invitrogen, Carlsbad, CA, USA). DNA bisulfite modification was performed with an EZ DNA Methylation-Gold Kit (Zymo Research, Orange, CA, USA) according to the manufacturer’s instructions. The modified DNA was used as a template for MethyLight.

### TaqMan probe-based quantitative methylation-specific PCR (MethyLight)

The methylation levels of TUBB1 promoter were detected using MethyLight in all participants. The promoter of TUBB1 was delineated with the website (https://www.ncbi.nlm.nih.gov/) and the sequence was transformed on another website (https://www.urogene.org/methprimer/). The genome coordinates of TUBB1 are hg38, chr20:60802540-60809767. The promoter region was considered to be the upstream 2,000 bp region of its transcription start site, where one CpG island was found from 1,025 to 1,131 bp (Fig. S1). The primers and probes were designed at the CpG island region using oligo7 (OLIGO 1267 Vondelpark ColoradoSprings, CO 80907, USA). The specific primers and probe sequences for gene promoters are listed in Table 1. We used a 10 µL volume MethyLight reaction system, including 5 µL MethyLight Master Mix (consisting of HotStarTaq Plus DNA Polymerase, EpiTect Probe PCR Buffer, and dNTP mix [dATP, dCTP, dGTP, dTTP]), 0.4 µL forward primer, 0.4 µL reverse primer, 0.2 µL probe, 2 µL nuclease-free water, and 2 µL modified DNA. The reaction was cycled using the following conditions: 95°C for 15 min and 45 cycles of 95°C for 15 s and 60°C for 60 s. SSSI methylase and bisulfite-modified human control DNA (QIAGEN, Hilden, Germany) served as the reference for methylation and β-actin was used as a normalization control. The results of MethyLight data were indicated by percentage of methylated reference (PMR) (31). PMR = 100% × 2^(−[Delta Ct (target gene in sample − control gene in sample) − Delta Ct (100% methylated target in reference sample − control gene in reference sample)]) (32).

**TABLE 1:** Primer and TaqMan probe sequences used to amplify bisulfite-converted DNA and quantitative real-time polymerase chain reaction (RT-qPCR)

Gene	Forward primer sequence (5′−3′)	Reverse primer sequence (5′−3′)	Probe oligo sequence	Amplicon size (bp)
MethyLight				
TUBB1	GGTATGATTTCGGTTTATTATAATTT	CTTAAAACCGAATATAATAACTCACT	ACCAACATAACGAAACCCCATCTCTT	248
β-actin	TGGTGATGGAGGAGGTTTAGTAAGT	AACCAATAAAACCTACTCCTCCCTTAAA	ACCACCACCCAACACACAATAACAAACACA	133
RT-qPCR				
TUBB1	GGAGATGATTGGTGAGGAACACG	GGTTCTAGGTCCACCAAGACTG		147
β-actin	ATGGGTCAGAAGGATTCCTATGTG	CTTCATGAGGTAGTCAGTCAGGTC		435

### RNA extraction and quantitative real-time polymerase chain reaction

Total RNA of PBMCs was extracted using TRIzol Reagent (Invitrogen, Carlsbad, CA, USA). We used a reverse transcription kit to convert RNA into cDNA following the manufacturer’s instructions (ThermoFisher, Waltham, USA). The expression levels of TUBB1 and β-actin mRNA were quantified using real-time PCR. The total reaction volume was 10 µL, consisting of 5 µL of TB Green premix (Takara, Shiga, Japan), 3 µL of nuclease-free water, 0.5 µL of forward primer, 0.5 µL of reverse primer, and 1 µL of cDNA. The PCR cycling was performed using a thermocycler from Analytik Jena (Germany), with conditions of denaturation at 95°C for 30 s, followed by 40 cycles of 95°C for 5 s, 55°C for 30 s, and 72°C for 60 s. The primer sequences are shown in Table 1. The comparative method (2^−ΔΔCt^) was utilized.

### Statistical analysis

Quantitative variables are expressed as the median (centile 25 and centile 75) and categorical variables are expressed as frequency (percentage). We used the Mann-Whitney U-test and the Kruskal-Wallis H-test to compare quantitative variables and the chi-square test to analyze categorical variables. The Spearman’s rank correlation test was used to determine the correlation between the TUBB1 promoter methylation level and clinical data. Independent risk factors for HBeAg SC were analyzed by binary logistic regression analysis with multivariate stepwise regression. The receiver operating characteristics (ROC) curve was constructed to obtain the area under the curve (AUC) and the best cut-off value was calculated, corresponding to the highest Youden index. Statistical analyses were performed using SPSS (version 26.0) and GraphPad Prism (version 9.5.1). Two-tailed *P* < 0.05 was considered statistically significant.

## RESULTS

### Study population

A total of 271 participants were enrolled in this study, including 239 patients with CHB and 32 HCs. Among the patients with CHB, 145 patients were HBeAg-positive, and 94 patients were HBeAg-negative.

Compared to patients with negative HBeAg, patients with positive HBeAg were younger and had higher ALT, AST, GGT, AFP levels and lower ALB levels. Serum HBV DNA was more likely to be detected in patients with positive HBeAg, while HBV DNA and HBsAg levels in patients with positive HBeAg were significantly higher than those with negative HBeAg results (Table 2).

**TABLE 2:** Characteristics of study participants^*c*^^,^^*d*^

	HC (*n* = 32)	HBeAg− (*n* = 94)	HBeAg+ (*n* = 145)	*P* value^*a*^	*P* value^*b*^
Male, n (%)	19 (59.38)	63 (67.02)	88 (60.69)	0.562	0.322
Age (years)	49.00 (37.25–55.75)	45.00 (37.00–52.25)	40.00 (33.50–52.00)	0.014	0.021
ALT (U/L)	17.00 (13.00–27.50)	22.00 (16.00–33.50)	31.00 (18.00–82.00)	<0.001	0.002
AST (U/L)	17.00 (15.00–24.50)	22.00 (19.00–30.00)	28.00 (29.50–51.50)	<0.001	0.004
AKP (U/L)	66.00 (58.00–73.00)	79.50 (65.00–98.25)	85.00 (68.00–109.00)	<0.001	0.145
GGT (U/L)	21.00 (15.75–31.00)	20.00 (16.00–32.75)	29.00 (16.00–62.00)	0.020	0.010
TBIL (µmol/L)	10.30 (8.02–12.10)	11.85 (9.10–16.93)	11.50 (8.35–17.45)	0.053	0.547
ALB (g/L)	46.85 (45.25–49.83)	47.80 (45.88–50.20)	45.40 (42.45–48.70)	0.001	<0.001
Cr (µmol/L)	72.00 (56.00–81.00)	71.00 (58.00–80.00)	68.00 (54.00–77.00)	0.310	0.169
BUN (mmol/L)	5.50 (4.34–5.80)	4.90 (4.10–5.70)	4.60 (3.73–5.76)	0.087	0.119
PLT (10^9^/L)	246.00 (213.00–276.75)	193.50 (131.25–237.25)	197.50 (147.50–242.25)	0.002	0.332
INR	0.91 (0.89–0.93)	1.00 (0.94–1.18)	1.05 (0.97–1.22)	<0.001	0.564
PTA	117.00 (111.00–121.00)	98.00 (70.75–110.75)	91.00 (70.00–107.25)	<0.001	0.666
AFP (ng/mL)	3.72 (2.59–5.06)	2.26 (1.63–3.68)	3.41 (2.31–6.60)	<0.001	<0.001
HBsAg (IU/mL)	NA	1,858.60 (532.52–5,240.70)	6,051.54 (2,159.82–28,417.58)		<0.001
Detectable HBV DNA, n (%)	NA	39 (41.49)	112 (77.24)		<0.001
Log_10_ [HBV DNA]	NA	3.47 (3.11–4.48)	6.78 (4.41–8.26)		<0.001
PMR (%)	11.01 (8.85–13.43)	13.96 (11.41–16.17)	15.83 (13.26–18.33)	<0.001	<0.001
					

^*a*^ *P* value is for the test of differences among HCs, HBeAg-negative, and HBeAg-positive.

^*b*^ *P* value is for the test of differences between HBeAg-negative and HBeAg-positive.

^*c*^ Quantitative variables are expressed as the median (25th percentile and 75th percentile). Categorical variables are expressed as the number (%).

^*d*^ HCs, healthy controls; HBeAg, hepatitis B e antigen; ALT, alanine aminotransferase; AST, aspartate aminotransferase; AKP, alkaline phosphatase; GGT, γ-glutamyl transpeptidase; TBIL, total bilirubin; ALB, albumin; Cr, creatinine; BUN, blood urea nitrogen; PLT, blood platelet; INR, international normalized ratio; PTA, prothrombin time activity; AFP, alpha-fetoprotein; HBsAg, hepatitis B s surface antigen; HBV, hepatitis B virus; NA, not available; PMR, percentage of methylated reference.

### Hypermethylation of the TUBB1 promoter and low mRNA expression of TUBB1 in patients with positive HBeAg

**Fig 1:**
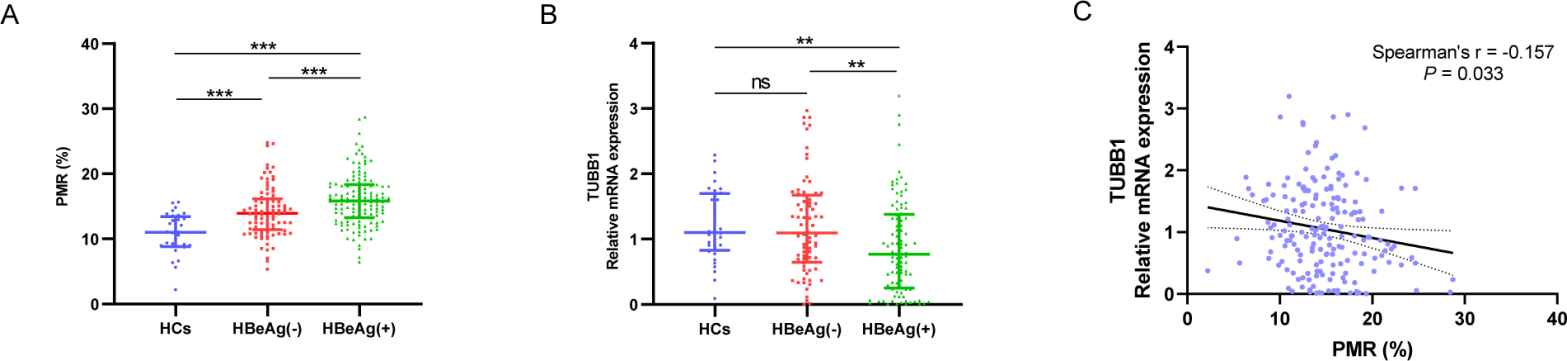
Relationships contrasting promoter methylation and mRNA expression of TUBB1 in PBMCs among different groups of participants. (**A**) TUBB1 methylation levels (PMR) in PBMCs of HCs, HBeAg-negative, and HBeAg-positive groups (**P < 0.01; ***P < 0.001). (**B**) TUBB1 mRNA levels in PBMCs of HCs, HBeAg-negative, and HBeAg-positive groups (ns, not significant). (**C**) Relationships between the TUBB1 promoter methylation levels and mRNA levels in PBMCs.

The methylation status of the TUBB1 promoter in PBMC was evaluated using MethyLight and expressed as PMR. Figure 1A depicts the methylation level of the TUBB1 promoter in HCs, HBeAg-negative, and HBeAg-positive groups, respectively. The TUBB1 methylation levels in patients with positive HBeAg (median 15.83, interquartile range 13.26–18.33) were significantly higher than that in those with negative HBeAg (median 13.96, interquartile range 11.41–16.17; *P* < 0.001) and HCs (median 11.01, interquartile range 8.85–13.43; *P* < 0.001). In addition, the TUBB1 methylation levels of HBeAg-negative participants were significantly higher than HCs (*P* < 0.001).

Since methylation is a prevalent mechanism that regulates transcription, we examined the expression pattern of TUBB1 mRNA in PBMCs from HCs and patients with HBeAg-negative, as well as HBeAg-positive (Fig. 1B). The mRNA expression level of TUBB1 in the HBeAg-positive group was significantly lower than that in the HBeAg-negative (*P* = 0.004) and HC (*P* = 0.005) groups. There was no difference in the mRNA level of the TUBB1 between the HBeAg-negative group and the HC group (*P* = 0.550).

To further elucidate the association between the methylation level of the TUBB1 promoter and its mRNA expression level, we conducted a Spearman’s rank correlation analysis. Our results indicated a significant, albeit weak, negative correlation between the methylation status of the TUBB1 promoter and its mRNA expression (Spearman’s r = −0.157, *P* = 0.033; Fig. 1C).

### The TUBB1 promoter methylation level was related to the phases of CHB

**Fig 2:**
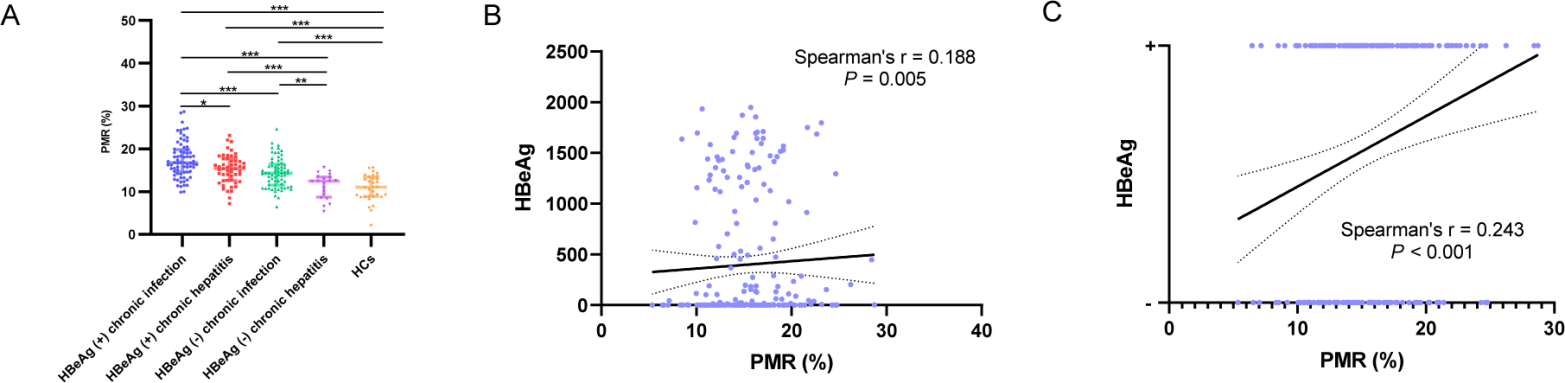
The methylation levels of the TUBB1 promoter in various phases of CHB and the association between TUBB1 promoter methylation and HBeAg. (**A**) The methylation levels of the TUBB1 promoter (PMR) in various phases of CHB and HCs are shown (*P < 0.05; **P < 0.01; ***P < 0.001). (**B**) The association between TUBB1 promoter methylation and HBeAg. (**C**) HBeAg status was significantly correlated with TUBB1 promoter methylation values.

All the patients with CHB were selected into the different phases according to the 2017 EASL guidelines (5). There were 85 cases in HBeAg-positive chronic infection (immune tolerant), 53 in HBeAg-positive chronic hepatitis (immune [re]active), 70 in HBeAg-negative chronic infection (inactive carrier state), and 22 in HBeAg-negative chronic hepatitis (immune-active or reactivation). Besides, nine patients were in the gray zone. The TUBB1 promoter methylation levels showed a sequential decrease in the order of HBeAg-positive chronic infection, HBeAg-positive chronic hepatitis, HBeAg-negative chronic infection, and HBeAg-negative chronic hepatitis. Among the participants with positive HBeAg, those who had raised ALT during the HBeAg-positive chronic hepatitis phase exhibited lower TUBB1 promoter methylation levels than those with normal ALT, who were in the HBeAg-positive chronic infection phase. Among patients with normal ALT, those in the HBeAg-positive chronic infection phase had higher methylation levels compared to those in the HBeAg-negative chronic infection phase (Fig. 2A).

On the other hand, the relationship between the TUBB1 promoter methylation and clinicopathology was analyzed in patients with CHB. As shown in Table 3 and Fig. 2B, we found that PMR was significantly and positively correlated to HBeAg. Furthermore, HBeAg status (positive or negative) was significantly correlated with PMR values, as demonstrated by Spearman’s correlation analysis (Spearman’s r = 0.243, *P* < 0.001; Fig. 2C). However, there was no statistically significant correlation between PMR and other clinical indices.

**TABLE 3:** The relationships between PMR and clinical data in CHB patients

	Spearman’s r	*P* value
PMR (%)		
Age (years)	−0.065	0.339
Sex	0.027	0.686
ALT (U/L)	−0.010	0.879
AST (U/L)	−0.040	0.554
AKP (U/L)	0.022	0.739
GGT (U/L)	−0.001	0.983
TBIL (µmol/L)	−0.069	0.310
ALB (g/L)	−0.061	0.370
Cr (µmol/L)	0.007	0.921
BUN (mmol/L)	0.048	0.492
PLT (10^9^/L)	0.045	0.508
INR	0.091	0.433
PTA	−0.186	0.136
AFP (ng/mL)	0.082	0.240
HBsAg (IU/mL)	0.037	0.592
HBeAg (IU/mL)	0.188	0.005^*a*^
Detectable HBV DNA	0.020	0.766
Log_10_ [HBV DNA]	−0.076	0.371

^*a*^ *P* < 0.05.

### Relatively lower TUBB1 promoter methylation level as a predictor for HBeAg SC in patients with positive HBeAg

**Fig 3:**
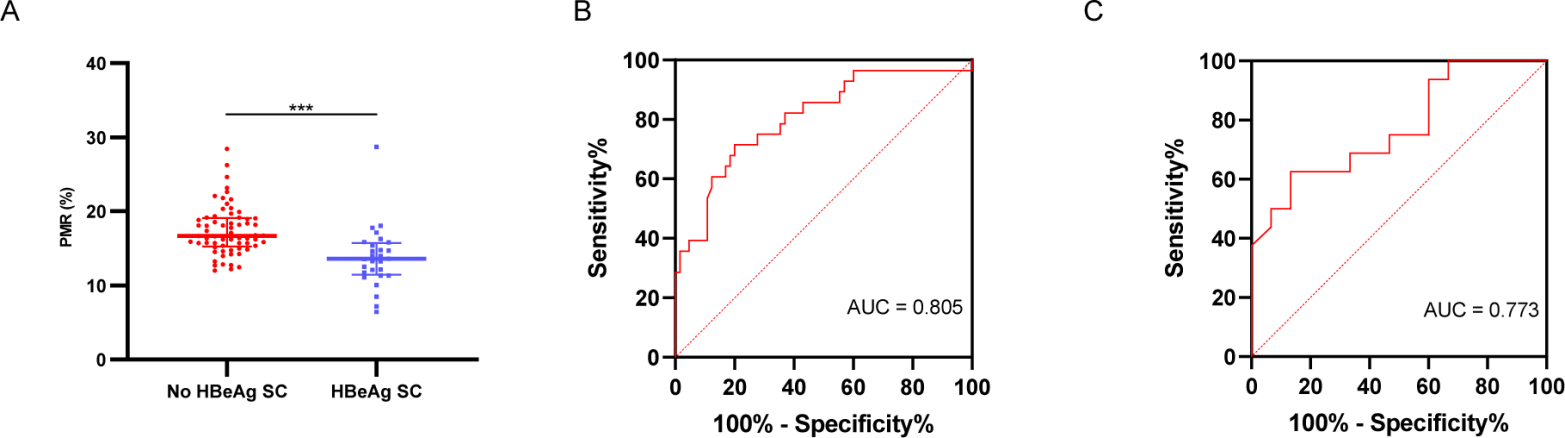
The TUBB1 promoter methylation and HBeAg SC. (**A**) Comparisons of the methylation levels of the TUBB1 promoter (PMR) between patients who achieved HBeAg SC and those who did not (***P < 0.001). (**B**) ROC curve of PMR for predicting HBeAg SC in HBeAg-positive patients. The optimal cut-off value was 14.80%, with an AUC of 0.805 (95% CI: 0.704–0.907), sensitivity of 80.00%, specificity of 71.43%, and *P* < 0.001. (**C**) ROC curve of PMR for predicting HBeAg SC in patients with HBeAg-positive and elevated ALT (AUC 0.773, 95% CI 0.609–0.937, *P* = 0.001).

Of the 93 patients with positive HBeAg who completed the 72-week follow-up, 28 developed HBeAg SC, while 65 did not. As shown in Table 4 and Fig. 3A, the TUBB1 methylation levels were significantly lower in patients with HBeAg SC than in those without HBeAg SC. Besides, compared with patients without HBeAg SC, patients with HBeAg SC contained more males and had significantly higher ALT and AST. There was no significant difference between the two groups in terms of either HBV-related indices or treatment.

**TABLE 4:** Comparison of characteristics between participants with and without HBeAg seroconversion^*a*^

	No HBeAg SC (*n* = 65)	HBeAg SC (*n* = 28)	*P* value
PMR (%)	16.69 (15.225–19.11)	13.60 (11.44–15.71)	<0.001
Male, n (%)	36 (55.38)	22 (78.57)	0.034
Age (years)	39.00 (34.00–52.00)	47.00 (37.50–55.00)	0.160
ALT (U/L)	23.00 (17.00–45.50)	52.50 (26.50–96.00)	0.017
AST (U/L)	25.00 (19.00–48.00)	35.50 (25.00–51.75)	0.023
AKP (U/L)	85.00 (68.00–105.00)	93.00 (70.00–112.50)	0.691
GGT (U/L)	24.00 (13.50–61.00)	34.00 (24.00–75.00)	0.050
TBIL (µmol/L)	10.00 (8.15–16.60)	12.35 (8.33–19.55)	0.995
ALB (g/L)	45.70 (42.70–49.20)	45.65 (43.45–49.20)	0.782
Cr (µmol/L)	65.00 (51.50–79.00)	68.00 (61.00–76.00)	0.087
BUN (mmol/L)	4.70 (3.60–5.50)	4.40 (4.10–5.80)	0.580
PLT (10^9^/L)	202.50 (147.50–252.75)	176.00 (104.75–225.75)	0.083
INR	1.08 (0.98–1.30)	1.06 (0.98–1.19)	0.370
PTA	86.00 (62.00–103.00)	97.00 (77.25–105.50)	0.289
AFP (ng/mL)	2.89 (2.12–6.64)	3.70 (2.59–6.46)	0.198
HBsAg (IU/mL)	5,512.90 (2,062.85–30,843.88)	2,375.13 (787.79–16,535.87)	0.055
HBeAg (IU/mL)	202.97 (24.51–1,339.60)	278.97 (2.09–1,097.85)	0.189
Detectable HBV DNA, n (%)	51 (78.46)	19 (67.86)	0.277
Log_10_ [HBV DNA]	5.62 (3.33–8.17)	6.96 (5.50–8.18)	0.280
Treatment			
TDF, n (%)	28 (43.08)	8 (28.57)	0.409
ETV, n (%)	28 (43.08)	16 (57.14)	
PEG-IFN+NAs, n (%)	2 (3.08)	2 (7.14)	
No therapy, n (%)	7 (10.77)	2 (7.14)	

^*a*^ TDF, tenofovir disoproxil fumarate; ETV, entecavir; PEG-IFN, peginterferon; NAs, nucleos(t)ide analogs.

To identify the significant factors that may affect the HBeAg SC, binary logistic regression analysis was performed (Table 5). A univariate analysis showed that PMR, gender, ALT, AST, and GGT were significant factors for predicting HBeAg SC with *P* < 0.05 and they were selected into the multivariate binary logistic regression model with stepwise analysis. As a result, PMR representing the methylation level of the TUBB1 promoter was a significant independent predictor for HBeAg SC (odds ratio [OR] = 0.683, 95% CI 0.553–0.845, *P* < 0.001). In addition, elevated ALT levels (≥40 U/L) were also demonstrated to be independent factors influencing HBeAg SC. Besides, based on the ROC curve analysis, the optimal cut-off value of PMR was <14.80% for predicting the occurrence of HBeAg SC (AUC = 0.805, 95% CI 0.704–0.907, sensitivity 80.00%, specificity 71.43%, *P* < 0.001; Fig. 3B). Specifically, the TUBB1 promoter methylation exhibited statistically significant predictive ability for HBeAg SC in HBeAg-positive patients with elevated ALT (AUC = 0.773, 95% CI 0.609–0.937, sensitivity 62.50%, specificity 88.67%, *P* = 0.001) (Fig. 3C).

**TABLE 5:** Univariate and multivariate analysis of HBeAg seroconversion in patients with HBeAg-positive CHB^*a*^

Variable	Univariate analysis		Multivariate analysis	
	OR (95% CI)	*P* value	OR (95% CI)	*P* value
PMR (%)	0.699 (0.578–0.846)	0.006	0.683 (0.553–0.845)	<0.001
Male	2.954 (1.058–8.246)	0.039		
Year (≥40)	2.177 (0.859–5.520)	0.101		
ALT (≥40 U/L)	4.444 (1.727–11.435)	0.002	4.951 (1.670–14.675)	0.004
AST (≥33 U/L)	3.771 (1.486–9.574)	0.005		
GGT (≥28.5 U/L)	3.304 (1.270–8.591)	0.014		
Detectable HBV DNA	0.580 (0.215–1.559)	0.280		
HBeAg (IU/mL)	1.000 (0.999–1.000)	0.371		
HBsAg (IU/mL)	1.000 (1.000–1.000)	0.057		

^*a*^ OR, odds ratio; CI, confidence interval.

## DISCUSSION

Our study showed that the TUBB1 promoter methylation levels were significantly higher in HBeAg-positive patients than in HBeAg-negative patients or in HCs. The methylation degree of the TUBB1 promoter gradually declined during the four phases of CHB. There was a weak but significant positive correlation between the TUBB1 promoter methylation level and the quantitative value of HBeAg. We found that TUBB1 promoter methylation and elevated ALT were independent factors for the achievement of HBeAg SC. Besides, the mRNA levels of TUBB1 were obviously lower in the HBeAg-positive group than in the HBeAg-negative group and HCs. The methylation status of the TUBB1 promoter exhibited a weak but significant negative correlation with the mRNA expression.

In our study, we observed hypermethylation of the TUBB1 promoter and, on the contrary, lower TUBB1 mRNA expression in the HBeAg-positive group compared to the HBeAg-negative group and HCs. Meanwhile, the methylation levels of the TUBB1 promoter were significantly inversely associated with the mRNA levels of TUBB1 (Spearman’s r = −0.157, *P* = 0.033). Previous studies have shown that CpG islands function as molecular switches, suppressing gene expression when methylated (33–35). It is reasonable to speculate that the methylation of the TUBB1 promoter inhibits the expression of TUBB1 mRNA to some degree. The weak correlation between promoter methylation and mRNA expression levels is likely attributable to the multifaceted nature of gene regulation. While promoter methylation is a recognized mechanism of gene silencing, mRNA expression is additionally modulated by multiple other factors, such as histone modifications, transcription factor activity, non-coding RNAs, chromatin architecture, and nuclear organization. Therefore, we did not delve into the potential molecular mechanisms in this research. Alternatively, we focused on the clinical significance of quantified methylation.

The PBMCs are mainly composed of different kinds of immune cells, including lymphocytes (T cells, B cells, and NK cells), monocytes, and dendritic cells. Several studies have already demonstrated that immune-metabolism disorder of liver tissue triggered by HBV exacerbation might result in the alteration of PBMCs (36, 37). Meanwhile, several previous studies also proved that aberrant DNA methylation status of PBMCs existed in patients with hepatitis B virus infection (32, 38). While PBMC methylation doesn’t directly match liver methylation, it’s a valuable indicator of the body’s overall response to chronic HBV and the immune-metabolic stress from the liver. Therefore, in our study, TUBB1 promoter methylation in PBMCs serves as a promising non-invasive biomarker for assessing disease status and predicting clinical outcomes, such as HBeAg seroconversion.

Given the intricate pathophysiology of HBV, the history and phases of infection continue to be under ongoing investigation (39). According to the 2017 EASL guidelines (5), the natural progression of chronic HBV infection can be broadly categorized into four phases: HBeAg-positive chronic infection (immune-tolerant phase), HBeAg-positive chronic hepatitis (immune [re]active phase), HBeAg-negative chronic infection (inactive carrier state phase), and HBeAg-negative chronic hepatitis (immune-active phase or HBeAg-negative disease). Specifically, patients within the HBeAg-positive chronic infection phase and the HBeAg-negative chronic infection phase maintain normal ALT levels. In contrast, those in the HBeAg-positive chronic hepatitis phase and the HBeAg-negative chronic hepatitis phase exhibit elevated ALT levels. CHB exhibits a non-linear clinical course and not all patients go through every phase (4, 5). Therefore, the phases of CHB and TUBB1 promoter methylation cannot be used for correlation analysis and ordinal logistic regression analysis. In our study, the methylation levels of the TUBB1 promoter tended to differ across the four phases, associated with different clinical genotype. Among HBeAg-positive patients, those who experienced an elevation in ALT levels during the HBeAg-positive chronic hepatitis phase exhibited lower levels of TUBB1 promoter methylation compared to patients with normal ALT values who were in the HBeAg-positive chronic infection phase. This phenomenon is consistent with our subsequent observation that patients who achieved HBeAg SC had higher ALT levels and lower TUBB1 promoter methylation compared to those who did not lose HBeAg. Meantime, TUBB1 promoter methylation was significantly positively correlated with the quantitative value of HBeAg. It could be inferred that PMR, representing the methylation level of the TUBB1 promoter, could reflect the clinical progression of CHB patients.

Huang et al. found that quantitative HBeAg and detectable baseline HBV DNA could predict the clearance of HBeAg in patients treated with pegylated interferon (40). Buster’s study showed that a higher ALT, low HBV DNA levels, female gender, older age, and an absence of previous interferon therapy were independent predictors of HBeAg SC in patients treated with peginterferon-alfa (41). In the studies conducted by Huang and Buster, patients were primarily treated with peginterferon, which raises concerns regarding selection bias due to the potential side effects and the limited applicability of this treatment to certain populations when investigating the predictive factors associated with HBeAg SC. However, our study is more closely aligned with actual clinical scenarios, and elevated ALT levels have been identified as independent predictive factors associated with HBeAg SC as well. In our study, however, there was no difference in HBV-related indicators between patients who had achieved HBeAg SC and those who had not. Besides, in our study, the HBeAg SC group comprised more male patients. Many other studies are consistent with our results (42–44).

However, there are still several limitations in our study. Firstly, it is a single-center study with a small sample size. Compared with lost patients, those who are willing to be reviewed are likely to have stronger treatment intention and follow the doctor’s advice more regularly, potentially introducing selection bias. Secondly, due to the difficulty in obtaining biopsy samples, we were unable to analyze the intrahepatic methylation status of TUBB1 in the studied patients. Furthermore, the precise molecular mechanisms by which TUBB1 is involved in the natural history of CHB and its role in HBeAg SC have not been further investigated.

### Conclusion

In conclusion, elevated TUBB1 promoter methylation levels in PBMCs are significantly associated with HBeAg-positive status and may serve as a promising non-invasive biomarker for predicting HBeAg SC.

## Data Availability

The data presented in this study are included in the article. Further inquiries can be directed to the corresponding author.

## SUPPLEMENTAL MATERIAL

Figure S1 spectrum.01344-25-s0001.pdf CpG island in the promoter of TUBB1.

ASM does not own the copyrights to Supplemental Material that may be linked to, or accessed through, an article. The authors have granted ASM a non-exclusive, world-wide license to publish the Supplemental Material files. Please contact the corresponding author directly for reuse.
