# Supplementary figures and images for: TUBB1 promoter methylation is a promising biomarker for predicting HBeAg seroconversion in chronic hepatitis B
Source: Microbiol Spectr. 2025 Oct 9;13(11):e01344-25. doi: 10.1128/spectrum.01344-25 (PMC12584639; doi:10.1128/spectrum.01344-25)

**Supplementary Figure S1 The CpG island in the promoter of TUBB1**

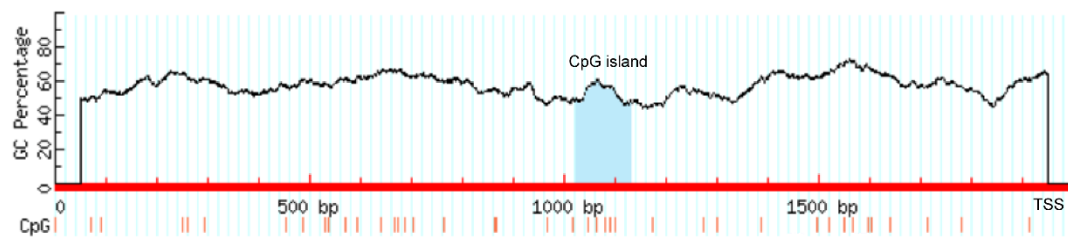

Supplement: Figure S1 — CpG island in the promoter of TUBB1. [file spectrum.01344-25-s0001.pdf]
